# Fat gain with physical detraining is correlated with increased glucose transport and oxidation in periepididymal white adipose tissue in rats

**DOI:** 10.1590/1414-431X20154356

**Published:** 2015-05-26

**Authors:** R.A.L. Sertié, S. Andreotti, A.R.G. Proença, A.B. Campaña, F.B. Lima

**Affiliations:** 1 Departamento de Fisiologia e Biofísica, Instituto de Ciências Biomédicas, Universidade de São Paulo, São Paulo, SP, Brasil; 2 Laboratório de Biotecnologia, Faculdade de Ciências Aplicadas, Universidade Estadual de Campinas, Limeira, SP, Brasil

**Keywords:** Physical detraining, Adipocytes, Glucose uptake, Glucose oxidation, Lipogenesis

## Abstract

As it is a common observation that obesity tends to occur after discontinuation of exercise, we investigated how white adipocytes isolated from the periepididymal fat of animals with interrupted physical training transport and oxidize glucose, and whether these adaptations support the weight regain seen after 4 weeks of physical detraining. Male Wistar rats (45 days old, weighing 200 g) were divided into two groups (n=10): group D (detrained), trained for 8 weeks and detrained for 4 weeks; and group S (sedentary). The physical exercise was carried out on a treadmill for 60 min/day, 5 days/week for 8 weeks, at 50-60% of the maximum running capacity. After the training protocol, adipocytes isolated from the periepididymal adipose tissue were submitted to glucose uptake and oxidation tests. Adipocytes from detrained animals increased their glucose uptake capacity by 18.5% compared with those from sedentary animals (P<0.05). The same cells also showed a greater glucose oxidation capacity in response to insulin stimulation (34.55%) compared with those from the S group (P<0.05). We hypothesize that, owing to the more intense glucose entrance into adipose cells from detrained rats, more substrate became available for triacylglycerol synthesis. Furthermore, this increased glucose oxidation rate allowed an increase in energy supply for triacylglycerol synthesis. Thus, physical detraining might play a role as a possible obesogenic factor for increasing glucose uptake and oxidation by adipocytes.

## Introduction

Physical exercise is known as a factor that increases the rates of triacylglycerol (TAG) mobilization and oxidation, thereby leading to fatty mass reduction (1,2). With discontinuation of a training program (physical detraining), the body systems tend to gradually readjust the achievements acquired in many functional parameters to return to a previous condition seen in the sedentary state (3).

The consequences of physical detraining on the white adipose tissue (WAT) have not yet been fully explored. Positive correlations were reported between physical detraining and gain in visceral fatty mass, with increasing cardiac risk factors in obese children (4). One interesting study also showed an increase in adipose mass (retroperitoneal, urogenital, and mesenteric) in Sprague-Dawley rats trained for 8 weeks and detrained for 4 weeks, with or without a hyperlipidic diet (5). We previously demonstrated that physical detraining for a 4-week period in rats was enough time to thoroughly recover the adiposity for which growth had been refrained during an 8-week period of training due to, among other factors, an increase in the lipogenic capacity of these animals (6).

Lipogenesis is an endergonic reaction that requires energy for the formation of ATP from oxidation of substrates. This energy is then spent in binding to glycerol and fatty acid molecules to form TAG. As an important lipogenic substrate, glucose may be used for both ATP production and TAG synthesis. Because physical detraining is associated with increases in lipogenic capacity in isolated adipocytes, our aim in this study was to measure the rates of glucose uptake and oxidation to see whether they parallel the metabolic events closely related to lipogenesis.

## Material and Methods

### Animals

Male Wistar rats (45 days old, weighing 200 g) were obtained from the Animal Resources Center, Instituto de Ciências Biomédicas, Universidade de São Paulo, and maintained with free access to food and water under constant temperature (23±1°C) and lighting conditions (12-h/12-h light/dark cycle, lights on at 07:00 p.m.). The rats were divided into two groups (n=10): 1) group D (detrained), previously trained for 8 weeks after which the training program was discontinued and the rats remained untrained for the following 4 weeks; 2) group S, age-matched animals that remained sedentary throughout the 12-week period until the animals were euthanized by decapitation. The exercise was performed on a treadmill for 60 min/day, 5 days/week. The exercise intensity was 50-60% of the maximal running capacity (7). This study was approved by the Ethics Committee on Animal Research of the Instituto de Ciências Biomédicas under number 045 (page 31, Book 2).

### Procedures

*Isolation of adipocytes*. Epididymal fat was withdrawn, weighed, and minced into fine pieces that were transferred to a buffer containing type 1 collagenase for adipocyte isolation as described elsewhere (8).

*[^3^H]-2Deoxy-D-glucose ([^3^H]-2DG) uptake in isolated adipocytes*. The [^3^H]-2DG uptake rates were measured in the absence (basal) and presence (stimulated) of insulin at the maximum effective concentration (10 nM). Forty-microliter aliquots of adipose cell suspension (at 20% lipocrit) were pipetted into plastic tubes that contained or did not contain 2 µL of insulin, and incubated for 15 min at 37°C. Subsequently, 10 µL of [^3^H]-2DG (0.4 mM final concentration and 1850 Bq/tube) was added, and the uptake reaction was allowed to proceed for exactly 3 min. The test was terminated by the addition of 0.6 mM phloretin (250 µL, in Earle’s/HEPES buffer and 0.05% dimethylsulfoxide) at 4°C. The entrapped radiation was measured in a beta-counter (Tricarb 2100 TR; Packard Instruments, USA).

*D-[U-^14^C]-glucose oxidation (^14^CO_2_ production) test in isolated adipocytes*. Adipocytes (approximately 20% lipocrit in 50 µL) were placed in 17×100-mm polypropylene tubes containing Krebs/Ringer/phosphate/1% bovine serum albumin buffer (450 µL) with 2 mM glucose pH 7.45 at 37°C, saturated with a gas mixture (95% O_2_/5% CO_2_) and 5 µL of D-[U-^14^C]-glucose (2 mM and 1850 µCi/tube), with or without insulin (10 nM), and incubated for 60 min at 37°C. Subsequently, 8 N H_2_SO_4_ (0.2 mL) was added and the released ^14^CO_2_ was adsorbed on a paper filter embedded with 0.2 mL of ethanolamine. The radiation emitted was determined in a beta counter (Tricarb 2100 TR; Packard Instruments) (9).

### Statistical analysis

The means±SE of the individual data from each group were analyzed using Student's *t*-test, unpaired and parametric. The upper limit of significance for rejection of the null hypothesis was established at 5% (P<0.05).

## Results

**Figure 1:**
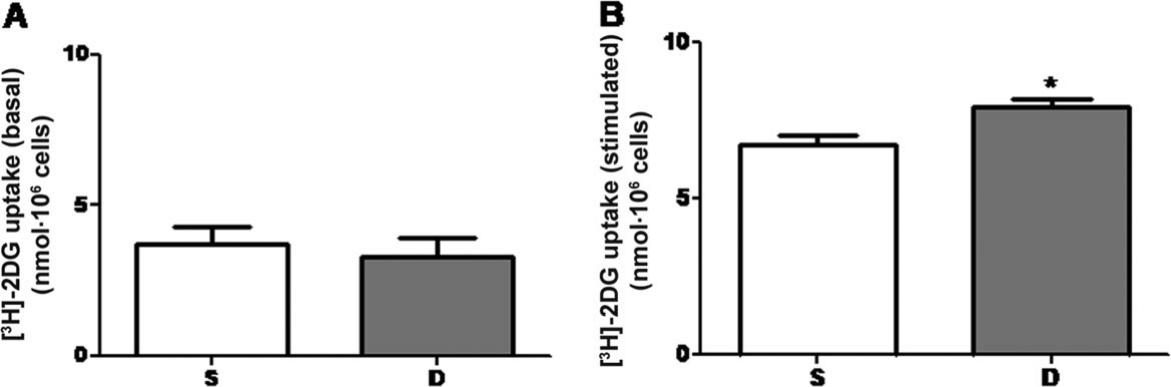
[^3^H]-2Deoxy-D-glucose ([^3^H]-2DG) uptake in isolated adipocytes. *Panels A and B* represent the supply of glucose by 10^6^ adipocytes isolated from the periepididymal fat of animals belonging to the sedentary (S) and detraining (D) groups. *A*, Basal (unstimulated) glucose uptake, and *B*, glucose uptake in the presence of insulin stimulation. *P<0.05, group S compared to group D (Student's *t*-test, n=10).

Adipocytes from detrained animals were more effective in taking up glucose (Figure 1B) when stimulated with insulin compared with those from the sedentary group (P<0.05). No differences among the groups were observed for the basal [^3^H]-2DG uptake rates (Figure 1A).

**Figure 2:**
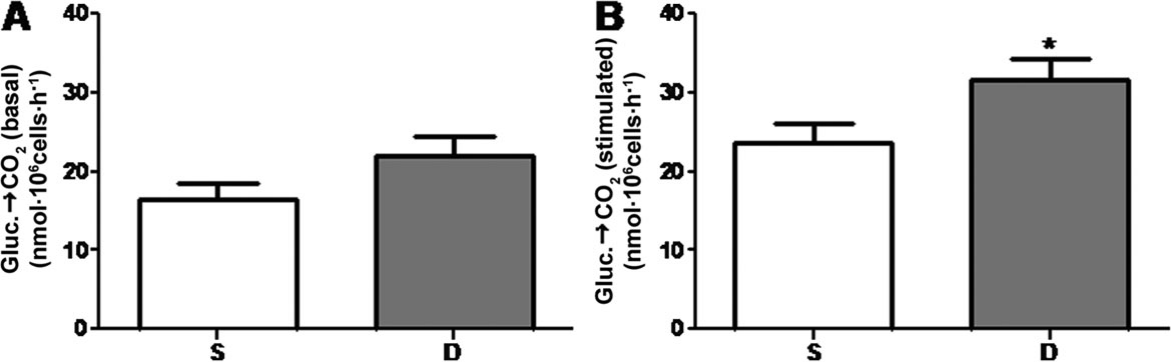
^14^CO_2_ released from D-[U-^14^C]-glucose in isolated adipocytes. *Panels A* and *B* represent the ^14^CO_2_ production from oxidized D-[U-^14^C]-glucose (Gluc.) by 10^6^ adipocytes isolated from the periepididymal fat of animals from the sedentary (S) and detrained (D) groups. *A*, Basal responses, and *B*, responses in the presence of insulin. *P<0.05, group S compared to group D (Student's *t*-test, n=10).

Similar results were found when the basal and insulin-stimulated rates of glucose oxidation were measured in isolated adipocytes from the two groups. Adipocytes from detrained rats were more responsive to insulin than those from sedentary animals (Figure 2B), and no differences were observed in the baseline responses (Figure 2A).

## Discussion

Previous studies have demonstrated that rats submitted to 10 weeks of swimming had increased GLUT1 and GLUT4 gene and protein expressions in the periepididymal fat (10). It is also recognized that physical training increases the insulin-dependent glucose transport in adipocytes (11). Physical training interruption did not cause an immediate loss of the acquired adaptations. Indeed, during a 4-week detraining period, the adipocytes sustained a more intense glucose transport ability in the presence of insulin, thus increasing the substrate availability for TAG production.

The reduction in adipose mass associated with physical exercise weakens production of tumor necrosis factor-α, interleukin-6, and plasminogen activator inhibitor-1, enhances adiponectin, and improves insulin sensitivity (12). Nevertheless, the increase in fat supplies along with physical detraining has been attributed to several factors, including increased insulin sensitivity and elevation of lipoprotein lipase activity (13,14). Conversely, it is known that when the fat mass grows, the production of pro-inflammatory adipokines intensifies, leading to insulin resistance (15). In the present study, this probably did not happen, at least in the periepididymal fat pad under examination, perhaps because the 4-week period of detraining was not sufficient to reverse the increase in the insulin-stimulated rate of glucose oxidation by the adipocytes of group D. We hypothesize that the increase in the lipogenic capacity of these cells (6) leads to an intensification of glucose oxidation to supply the amount of energy required to sustain fatty acid synthesis and esterification to glycerol-3-phosphate essential for TAG synthesis and storage. It is known that the increased demand for energy caused by exercise brings about an increase in fatty acid and glucose oxidation by adipocytes (16). Nonetheless, the phenomenon shown here to be strongly associated with detraining is unprecedented and deserves a deeper and more detailed investigation.

The glycolytic pathway generates pyruvate, which is transported to mitochondria, where it is transformed into acetyl-CoA under the action of pyruvate dehydrogenase (17). Although we cannot precisely identify the exact step in the oxidative pathway where the glucose metabolic route gained more efficiency, we observed that the adipocytes from the detrained animals reached a significant increment in their maximal capacity for metabolizing glucose.

In rats submitted to swimming for 4 months, the protein contents within the respiratory chain were increased, including cytochrome C oxidase subunit IV and cytochrome C oxidoreductase subunit I. In addition, the gene expressions of peroxisome proliferator-activated receptor-γ coactivator-1α (PGC1-α; the greatest regulator of mitochondrial biogenesis) and mitochondrial transcription factor A (a transcription factor that acts upstream in the cascade leading to activation of PGC1-α) were amplified, suggesting that physical training increased both the number and activity of mitochondria in the WAT (18). Thus, with training interruption, this adaptive mechanism generated during the previous training period could stay active, at least throughout 4 weeks following training interruption, creating a more favorable condition for ATP generation that, along with a greater supply of glucose inside the cells, leads to complete recovery of the animal's adipose mass, as previously described (6).

In conclusion, the present results may explain the body weight gain observed after a period of 4 weeks of physical detraining. The increased ability for transporting and oxidizing glucose developed by adipocytes when stimulated by insulin provides support to the idea that, to expand fat stores in the body, cells must obtain and metabolize more glucose. An important amount of this glucose must be directed to the tricarboxylic acid cycle for energy generation. Thus, physical training creates a favorable environment for building TAG molecules and consequently for replenishing the adipose mass at times of exercise discontinuation, which may work as an obesogenic factor.

## References

[1] Bukowiecki L, Lupien J, Follea N, Paradis A, Richard D, LeBlanc J (1980). Mechanism of enhanced lipolysis in adipose tissue of
exercise-trained rats. Am J Physiol.

[2] Hunter GR, Brock DW, Byrne NM, Chandler-Laney PC, Del Corral P, Gower BA (2010). Exercise training prevents regain of visceral fat for 1
year following weight loss. Obesity.

[3] Mujika I, Padilla S (2000). Detraining: loss of training-induced physiological and
performance adaptations. Part I: short term insufficient training
stimulus. Sports Med.

[4] Gutin B, Owens S, Okuyama T, Riggs S, Ferguson M, Litaker M (1999). Effect of physical training and its cessation on percent
fat and bone density of children with obesity. Obes Res.

[5] Yasari S, Dufresne E, Prud'homme D, Lavoie JM (2007). Effect of the detraining status on high-fat diet induced
fat accumulation in the adipose tissue and liver in female rats. Physiol Behav.

[6] Sertie RA, Andreotti S, Proenca AR, Campana AB, Lima-Salgado TM, Batista ML (2013). Cessation of physical exercise changes metabolism and
modifies the adipocyte cellularity of the periepididymal white adipose tissue in
rats. J Appl Physiol.

[7] Negrao CE, Moreira ED, Santos MC, Farah VM, Krieger EM (1992). Vagal function impairment after exercise
training. J Appl Physiol.

[8] Rodbell M (1964). Metabolism of isolated fat cells. Effects of hormones on
glucose metabolism and lipidis. J Biol Chem.

[9] Lima FB, Bao S, Garvey WT (1994). Biological actions of insulin are differentially
regulated by glucose and insulin in primary cultured adipocytes. Chronic ability
to increase glycogen synthase activity. Diabetes.

[10] Stallknecht B, Andersen PH, Vinten J, Bendtsen LL, Sibbersen J, Pedersen O (1993). Effect of physical training on glucose transporter
protein and mRNA levels in rat adipocytes. Am J Physiol.

[11] Ferrara CM, Reynolds TH, Zarnowski MJ, Brozinick JT, Cushman SW (1998). Short-term exercise enhances insulin-stimulated GLUT-4
translocation and glucose transport in adipose cells. J Appl Physiol.

[12] Aldhahi W, Hamdy O (2003). Adipokines, inflammation, and the endothelium in
diabetes. Curr Diab Rep.

[13] Applegate EA, Upton DE, Stern JS (1984). Exercise and detraining: effect on food intake,
adiposity and lipogenesis in Osborne-Mendel rats made obese by a high fat
diet. J Nutr.

[14] Craig BW, Thompson K, Holloszy JO (1983). Effects of stopping training on size and response to
insulin of fat cells in female rats. J Appl Physiol Respir Environ Exerc Physiol.

[15] Antuna-Puente B, Feve B, Fellahi S, Bastard JP (2008). Adipokines: the missing link between insulin resistance
and obesity. Diabetes Metab.

[16] Coyle EF, Jeukendrup AE, Wagenmakers AJ, Saris WH (1997). Fatty acid oxidation is directly regulated by
carbohydrate metabolism during exercise. Am J Physiol.

[17] Heinrich R, Melendez-Hevia E, Montero F, Nuno JC, Stephani A, Waddell TG (1999). The structural design of glycolysis: an evolutionary
approach. Biochem Soc Trans.

[18] Sutherland LN, Bomhof MR, Capozzi LC, Basaraba SA, Wright DC (2009). Exercise and adrenaline increase PGC-1{alpha} mRNA
expression in rat adipose tissue. J Physiol.

